# Skin Cancer and UV Exposure-Related Behaviors Among Appalachian and Non-Appalachian Adults

**DOI:** 10.13023/jah.0202.06

**Published:** 2020-04-15

**Authors:** Minal Patel, Katrina J. Serrano, Elise L. Rice, Chan L. Thai, Kelly D. Blake, Robin C. Vanderpool

**Affiliations:** Schroeder Institute, Truth Initiative; National Institute of Diabetes and Digestive and Kidney Diseases (NIDDK), National Institutes of Health; National Institute of Dental and Craniofacial Research, National Institutes of Health; Department of Communication, Santa Clara University; Behavioral Research Program, National Cancer Institute, National Institutes of Health; Behavioral Research Program, National Cancer Institute, National Institutes of Health

**Keywords:** UV exposure, sunscreen, tanning bed, skin cancer, Appalachian region

## Abstract

**Introduction:**

Appalachian communities experience elevated rates of cancer incidence and mortality relative to other regions in the U.S. Specifically, melanoma mortality rates are higher in Appalachia compared to the national average, despite comparable incidence rates.

**Purpose:**

To examine differences in self-reported history of skin cancer and prevalence of two UV exposure behaviors between Appalachian and non-Appalachian adults in a nationally representative sample.

**Methods:**

Data are from four cross-sectional cycles of the Health Information National Trends Survey (2011–2014) (N=14,451). We examined sunscreen use and tanning bed use, and self-reported history of melanoma and non-melanoma skin cancer. Descriptive and weighted multivariable analyses were conducted to examine sunscreen and tanning bed use, controlling for sociodemographic characteristics.

**Results:**

Approximately 8% of the study sample resided in Appalachia (n=1,015). Self-reported melanoma (0.6%) and non-melanoma (3.2%) skin cancer histories were low among Appalachians and did not differ statistically from non-Appalachians (*p*>0.05). Only 21.2% of Appalachians reported using sunscreen often or always when going outside for more than one hour on a warm, sunny day compared to 27.4% of non-Appalachians (*p*p=0.04), but there were no regional differences in tanning bed use (OR=1.48, p=0.23) when controlling for sociodemographics and general health status.

**Implications:**

Appalachians had comparable histories of self-reported melanoma and non-melanoma skin cancer but were less likely to report sunscreen use than non-Appalachians. Enhanced communication efforts to promote sunscreen use and other UV protection behaviors in Appalachia may be valuable.

## INTRODUCTION

The Appalachian region of the United States is a heterogeneous area that encompasses 13 states and includes approximately 25 million people.^1^ Appalachian residents experience notable health disparities, including elevated rates of cancer incidence and cancer mortality relative to the rest of the U.S. population,^2^,^3^ which have been attributed to limited access to health care (fewer clinics and lower rates of cancer screening); increased prevalence of high-risk health behaviors (higher rates of smoking and obesity); and lower socioeconomic status.^3^–^6^ In particular, melanoma mortality rates are 23% higher in Appalachia than the national average, despite comparable melanoma incidence rates.^7^ Incidence and mortality rates of non-melanoma skin cancers are not captured in cancer registries and claims data cannot provide region-specific prevalence estimates.

Exposure to ultraviolet (UV) light is the primary risk factor for most melanomas and non-melanoma skin cancers.^8^ Limiting skin damage from UV radiation, either from sunburns or exposure in tanning beds, can help to reduce skin cancer incidence and mortality. However, little is known regarding the use of sunscreen or tanning beds among Appalachian residents. A study of 90 undergraduates in Southern Appalachia found that 57% used sunscreen “at least some of the time they were exposed to the sun” and 27% reported using a tanning bed in the past week.^9^ In a national study, teenage girls living in non-metropolitan (rural) areas compared to metropolitan (urban) areas were 82% more likely to report indoor tanning use,^10^ despite the steady decline in tanning bed use nationally.^11^ Other important sun-protective behaviors include avoidance of the sun through the use of shade, hats, and long-sleeved clothing,^12^ although these behaviors have not been studied among Appalachian residents. Research examining sun-protective behaviors by urbanicity in the U.S. has demonstrated that rural residents were 33% less likely to wear sunscreen than urban residents.^13^ Aside from the limited studies described above, little is known about the UV-protection practices across the entire 13-state Appalachian region. The aim of this study was to examine differences in self-reported histories of melanoma and non-melanoma skin cancers and the prevalence of sunscreen use and tanning bed use between Appalachian and non-Appalachian adults in the U.S.

## METHODS

Data from four cycles of the fourth iteration of the Health Information National Trends Survey (HINTS 4), a nationally representative survey collected from 2011–2014, were combined for the analytic sample (N=14,451). The HINTS target population is the civilian non-institutionalized population of adults aged 18 or older in the U.S. The sampling frame consisted of residential addresses based on the U.S. Postal Service Computerized Delivery Sequence File. An equal probability sample of residential addresses were sent a self-administered paper instrument. Addresses located in Central Appalachia were oversampled in 2011 and 2012 (HINTS 4, Cycles 1 and 2) due to limited representation in prior iterations of HINTS, and high minority areas were oversampled in all four cycles of HINTS 4 to increase the precision of estimates for racial and ethnic populations.^14^ HINTS was approved by the Westat Institutional Review Board (IRB) and was deemed exempt from IRB review by the National Institutes of Health Office of Human Subjects Research Protections. Further details about HINTS development and methodology are documented elsewhere.^15^

Sunscreen use was assessed with one item: “When you are outside for more than one hour on a warm, sunny day, how often do you wear sunscreen?” Responses were dichotomized into “often or always,” vs. “sometimes, rarely, or never,” consistent with previous research.^13^,^16^ Tanning bed use was determined by responses to another item: “How many times in the past 12 months have you used a tanning bed or booth?” which were dichotomized to “no visits” versus “1 or more visits”. This question was not asked in Cycle 2 (October 2012–January 2013), resulting in a smaller sample (n=10,521) for analyses using this variable. Although other sun-protection behaviors of seeking shade and the use of hats and long-sleeved clothing were asked in Cycle 3, there was an insufficient sample of Appalachian residents, and therefore, these measures could not be included in the study. Cancer history was determined by self-reported prior diagnosis of melanoma or non-melanoma skin cancer. Sociodemographic variables included gender, age, race/ethnicity, income, and urbanicity (classified as urban or rural based on 2003 Rural–Urban Continuum Codes). Self-described general health status was also included as a covariate.

Descriptive and bivariate analyses were conducted to examine self-reported skin cancer history by residence (i.e., Appalachia or non-Appalachia). Weighted multivariable logistic regression analyses were conducted to examine sunscreen use and tanning bed use, controlling for sociodemographic characteristics and general health status. Analyses were completed in SAS 9.3 and SAS-callable SUDAAN version 11.0 to account for the complex sampling design. Jackknife replicate weights were employed to control for bias in variance estimates.

## RESULTS

Sample characteristics are presented in Table 1. Approximately 8% of the HINTS sample resided in Appalachia (n=1015). Approximately 87% of the Appalachian respondents were white compared to 65% of the non-Appalachian respondents; the overall ethnic/racial distribution was significantly different than the non-Appalachian sample (p<0.0001). Approximately 60% of the Appalachian sample had completed some college or technical training or higher, compared to 67% of the non-Appalachian sample (p=0.009). Other significant differences between Appalachian and non-Appalachian respondents included household income, general health status, and urbanicity (p<0.05).

Self-reported melanoma history among Appalachians was low (0.6%) and did not significantly differ from non-Appalachians (0.7%) (p=0.573) in the HINTS sample. Self-reported non-melanoma skin cancer histories also did not differ statistically between Appalachian and non-Appalachian adults (3.2% vs. 2.8%, respectively; p=0.462) in the HINTS sample. In terms of UV exposure-related behaviors, only 21.2% of Appalachian respondents reported using sunscreen often or always when going outside for more than one hour on a warm, sunny day compared to 27.4% of non-Appalachians (p=0.003). Additionally, 5.5% of Appalachian adults reported tanning bed use compared to 3.3% of non-Appalachians (p=0.025).

Table 2 presents the results of two multivariable logistic regression analyses assessing sunscreen use and tanning bed use, respectively. Among U.S. adults, Appalachians were less likely to use sunscreen “often or always” compared to non-Appalachians (OR=0.76, p=0.04), controlling for sociodemographics and general health status. In addition, women were more likely than men to report sunscreen use often or always (OR=2.20, p<0.0001) as were individuals with some college or technical training (OR=1.57, p=0.01) and a college degree or higher (OR=2.68, p<0.0001) compared with individuals with less than a high school degree. Annual household income and self-described health status were also associated with sunscreen use with higher income and better health associated with higher rates of sunscreen use. Non-Hispanic blacks (OR=0.21, p<0.0001) and individuals identifying as “Other” (OR=0.70, p=0.02) were less likely than non-Hispanic Whites to report sunscreen use. In examining differential use of tanning beds among U.S. adults, there were no significant differences between Appalachians and non-Appalachians when controlling for demographic characteristics and general health status (Table 2). In the full sample, women were significantly more likely than men to report tanning bed use (OR=5.10; p<0.0001) as were individuals with a household income of $100,000 or more (OR=2.34; p=0.04). Non-Hispanic blacks (OR=0.08, p<0.01) and Hispanics (OR=0.28, p<0.01) were less likely to report tanning bed use than non-Hispanic Whites.

## IMPLICATIONS

Consistent with findings from national surveillance data, the present analyses revealed Appalachian respondents were no more or less likely to report being diagnosed with melanoma compared to non-Appalachian U.S. adults. This study also revealed that self-reported non-melanoma skin cancer histories among Appalachians were comparable to non-Appalachians in the HINTS sample. One key behavioral difference also emerged: Appalachians indicated that they were less likely than non-Appalachians to use sunscreen regularly. This finding extends limited prior research on sunscreen use among younger adults in Appalachian Ohio,^9^ suggesting that lower use of sunscreen may be prevalent throughout the broader Appalachian region. This study’s strength is that it provides a probability-based examination of these factors from a national survey given that very limited representative data are available from Appalachian residents regarding skin cancer and related behaviors.

Although the current study provides a glimpse into challenges and opportunities for skin cancer prevention in Appalachia, many important questions remain. First, it should be noted that self-reported health histories (i.e., skin cancer diagnoses) are recognized for recall bias and are not as accurate as incidence data available from cancer registries. However, self-reported HINTS data do provide context for the UV behaviors assessed in the survey and allow for comparison across Appalachian and non-Appalachians in a way that registry data cannot. Our study revealed no geographic differences in tanning bed use when controlling for sociodemographics and general health status between Appalachians and non-Appalachians. Reductions in tanning bed use overall may explain why there were no statistically significant differences between Appalachian and non-Appalachian tanning bed use in multivariable analyses as use was relatively low across the U.S. with only 3.5% of the entire study sample reporting this behavior.^11^

Additionally, it should be noted that when adding covariates to the multivariable models, demographic covariates attenuated the observed relationship between Appalachian residence and sunscreen use, and accounted for the variation in tanning bed use, suggesting that demographic characteristics also are important predictors of UV-protection behaviors. Demographic differences between the Appalachian and non-Appalachian populations observed in our study indicate that individuals living in Appalachia were significantly more likely to be white and have lower socioeconomic status than non-Appalachians. These demographic characteristics may influence differences in sun-protection behaviors and need to be further examined. Additionally, the single item of sunscreen use does not account for other measures of sun-protection behaviors, such as wearing a hat, and the limited response scale may not optimally capture variance in sunscreen use. Sunscreen use alone does not capture the full picture regarding sun-protection behaviors. If individuals seek shade and/or wear long-sleeved clothing, sunscreen use may be lower, as individuals may be utilizing these other preventive behaviors instead.^12^ In order to fully understand sun-protection behaviors and UV-exposure among Appalachian residents, multiple sun-protection behaviors, along with sunbathing behaviors and sunburn prevalence, should be examined concurrently. Future studies may therefore consider other behaviors related to skin cancer prevention as well as additional stages of the cancer continuum. For example, future research may explore skin cancer screening rates and treatment options among residents of Appalachia.^2^ Lack of knowledge and self-efficacy may be additional barriers for practicing sun safety behaviors, such as sunscreen use, as seen among outdoor workers in Northern Mississippi.^17^ More research to fully understand potential barriers to practicing sun protective behaviors is important prior to intervention development.

Future research should evaluate awareness of skin cancer risk in a geographic region where other risk factors – especially tobacco use – have been the predominant foci of public health efforts. Although several interventions targeted towards Appalachian residents have focused on other health behaviors such as tobacco use, interventions targeted towards skin cancer have been limited. Lessons learned from smoking cessation programs among Appalachians, such as tailoring materials to be culturally appropriate, should be applied to interventions directed at limiting UV exposure.^18^ However, adapting and implementing cancer education interventions can be challenging in this region, and need to include key stakeholders and community participation for success.^19^ Given the results of this study, researchers and health practitioners have an opportunity to further examine other sun-protection behaviors in this underserved group, and can work at the community level to improve rates of sun-protective behaviors.

**SUMMARY BOX**

**What is already known on this topic?** Prior evidence of higher melanoma cancer mortality—despite comparable incidence—may suggest a shortage of resources for access to care for proper diagnosis and treatment of skin cancers. Considered within the larger social, economic, and cultural dimensions of Appalachia, these contextual factors underscore the importance of sun-protection behaviors.

**What is added by this report?** The present analyses suggest the need for further examination of multiple UV-protection–related behaviors among Appalachian residents.

**What are the implications for future research?** Enhanced communication efforts and interventions to promote sunscreen and other UV-protection behaviors among adults living in Appalachia may be particularly valuable.

## Figures and Tables

**Table 1:** Sample distributions for demographics and UV exposure related measures from the Health Information National Trends Survey (HINTS) 4, Cycles 1–4, 2011–2014*

	Appalachia		Non-Appalachia		Full Sample		
	*n*	%	*n*	%	*n*	%	*p-value*
**Age**							0.442
18–29	74	20.92	1051	20.15	1125	20.21	
30–49	248	33.28	3863	37.55	4111	37.21	
50–69	462	33.27	5691	30.39	6153	30.62	
70 +	196	12.54	2394	11.91	2590	11.96	
**Gender**							0.436
Male	381	46.48	5182	48.60	5563	48.43	
Female	610	53.52	7956	51.40	8566	51.57	
**Race/Ethnicity**							**<0.0001**
Hispanic	29	3.61	1994	15.96	2023	14.98	
White	693	86.58	7325	65.14	8018	66.84	
Black	110	6.35	1917	11.44	2027	11.03	
Other	45	3.46	882	7.47	927	7.15	
**Educational Attainment**							**0.009**
Less than high school	119	13.21	1206	11.80	1325	11.92	
HS graduate or equivalent	248	26.19	2681	21.09	2929	21.50	
Some college or technical training	285	34.14	3962	32.76	4247	32.87	
Bachelor’s degree or higher	329	26.46	5207	34.35	5536	33.71	
**Annual household income**							**0.006**
Less than $20,000	263	24.97	3077	21.46	3340	21.74	
$20,000 to <$35,000	164	15.71	2008	14.83	2172	14.90	
$35,000 to <$50,000	153	16.16	1898	14.23	2051	14.39	
$50,000 to <$75,000	173	19.96	2156	16.78	2329	17.04	
$75,000 to <$100,000	101	10.36	1545	12.95	1646	12.74	
$100,000 or more	133	12.85	2362	19.74	2495	19.18	
**General Health Status**							**0.013**
Excellent	98	9.33	1500	12.85	1598	12.57	
Very good	323	39.05	4594	36.08	4917	36.31	
Good	364	35.33	4790	36.60	5154	36.50	
Fair	150	11.97	1797	12.22	1947	12.20	
Poor	50	4.32	425	2.25	475	2.41	
**Urbanicity**							**<0.0001**
Urban	621	59.36	11653	85.51	12274	83.39	
Rural	394	40.64	1783	14.49	2177	16.61	
**Non-melanoma skin cancer diagnosis**							0.462
Yes	49	3.16	556	2.75	605	2.78	
No	862	96.84	11613	97.25	12475	97.22	
**Melanoma diagnosis**							0.573
Yes	11	0.56	141	0.69	152	0.68	
No	862	99.44	11613	99.31	12475	99.32	
**Sunscreen use**							**0.003**
Often or Always	237	21.21	3696	27.42	3933	26.91	
Sometimes, Rarely, or Never	974	78.79	9218	72.58	9955	73.09	
**Tanning bed visit**							**0.025**
Yes	35	5.45	244	3.29	279	3.47	
No	734	94.55	9565	96.71	10264	96.53	

* *n* is the unweighted sample size and the percentage (%) represents the weighted population estimate. *P*-values pertain to the results of unadjusted *X*^2^ tests comparing responses from Appalachians and non-Appalachians. Tanning bed visit was measured in Cycles 1, 3, and 4 of HINTS 4.

**Table 2:** Weighted, multivariable logistic regression depicting sunscreen use and tanning bed use among Appalachians compared to non-Appalachians*

	Model 1: Sunscreen Use			Model 2: Tanning Bed Use		
	OR	(95% Confidence Interval)	p-value	OR	(95% Confidence Interval)	p-value
**Appalachian Residence** *	**0.76**	**(0.59, 0.99)**	**0.04**	1.48	(0.78, 2.80)	0.23
**Age**	1.00	(1.00, 1.01)	0.62	**0.96**	**(0.94, 0.97)**	**<0.0001**
**Gender**						
Male	Reference	Reference	Reference	Reference	Reference	Reference
Female	**2.20**	**(1.92, 2.52)**	**<0.0001**	**5.10**	**(3.1, 8.41)**	**<0.0001**
**Race/Ethnicity**						
White	Reference	Reference	Reference	Reference	Reference	Reference
Black	**0.21**	**(0.16, 0.27)**	**<0.0001**	**0.08**	**(0.01, 0.41)**	**<0.01**
Hispanic	0.83	(0.66, 1.05)	0.13	**0.28**	**(0.13, 0.62)**	**<0.01**
Other	**0.70**	**(0.52, 0.96)**	**0.02**	0.37	(0.07, 1.88)	0.23
**Highest grade or level of schooling completed**						
Less than high school	Reference	Reference	Reference	Reference	Reference	Reference
HS graduate or equivalent	1.26	(0.87, 1.82)	0.23	1.65	(0.52, 5.16)	0.39
Some college or technical training	**1.57**	**(1.12, 2.20)**	**0.01**	1.57	(0.53, 4.63)	0.41
Bachelor’s degree or higher	**2.68**	**(1.89, 3.79)**	**<0.0001**	1.08	(0.36, 3.24)	0.9
**Annual household income**						
Less than $20,000	Reference	Reference	Reference	Reference	Reference	Reference
$20,000 to <$35,000	1.11	(0.83, 1.47)	0.49	1.31	(0.56, 3.03)	0.54
$35,000 to <$50,000	1.33	(0.99, 1.78)	0.06	1.83	(0.83, 4.04)	0.14
$50,000 to <$75,000	**1.56**	**(1.19, 2.03)**	**<0.01**	1.41	(0.67, 2.96)	0.36
$75,000 to <$100,000	**1.52**	**(1.11, 2.06)**	**0.01**	0.95	(0.43, 2.11)	0.89
$100,000 or more	**1.76**	**(1.32, 2.34)**	**<0.01**	**2.34**	**(1.03, 5.30)**	**0.04**
**General Health Status**						
Excellent	Reference	Reference	Reference	Reference	Reference	Reference
Very good	**0.76**	**(0.59, 0.96)**	**0.02**	0.60	(0.36, 0.99)	0.05
Good	**0.58**	**(0.46, 0.75)**	**<0.0001**	0.65	(0.40, 1.08)	0.09
Fair	**0.54**	**(0.39, 0.75)**	**<0.01**	**0.36**	**(0.15, 0.85)**	**0.02**
Poor	0.60	(0.30, 1.21)	0.15	0.06	(0.00, 1000)	0.79
**Urbanicity**						
Rural	Reference	Reference	Reference	Reference	Reference	Reference
Urban	1.19	(0.97, 1.46)	0.09	0.91	(0.57, 1.45)	0.68

* Significant associations are bolded. All demographic covariates presented above were included in both Models 1 and 2.
